# Effectiveness and Predictors of Outcome for Psychotherapeutic Interventions in Clinical Settings Among Adolescents

**DOI:** 10.3389/fpsyg.2021.628977

**Published:** 2021-02-16

**Authors:** Vera Gergov, Nina Lindberg, Jari Lahti, Jari Lipsanen, Mauri Marttunen

**Affiliations:** ^1^Department of Adolescent Psychiatry, Helsinki University Hospital, University of Helsinki, Helsinki, Finland; ^2^Department of Forensic Psychiatry, Helsinki University Hospital, University of Helsinki, Helsinki, Finland; ^3^Faculty of Medicine, University of Helsinki, Helsinki, Finland

**Keywords:** adolescents, psychotherapy, art and occupational therapies, clinical setting, naturalistic study, predictors, dropout

## Abstract

**Background:**

The aim of this study was to investigate the effectiveness of psychotherapeutic interventions for clinically referred adolescents, as well as to examine whether sociodemographic, clinical, or treatment-related variables and patients’ role expectations predict treatment outcome or are possible predictors of treatment dropout.

**Method:**

The study comprised 58 adolescents (mean age 14.2, 65.5% female) suffering from diverse psychiatric disorders referred to psychotherapeutic interventions conducted in outpatient care. The outcome measures, The Beck Depression Inventory, and the Clinical Outcomes in Routine Evaluation – Outcome Measure were filled in at baseline and at 3-, 6-, and 12-month follow-ups. Possible predictors were assessed at baseline.

**Results:**

The results indicate that the mean level of symptoms and psychological distress decreased during the treatment, most reduction occurring in the first 6 months. The frequency of treatment sessions was the strongest predictor of good outcome. Adolescents with a higher level of externalizing problems or lower level of expectations for their own active role in treatment seem to have a higher risk of dropping out.

**Conclusion:**

Offering intensive treatment for a shorter period might be the most efficient way to gain symptom reduction and decrease psychological distress in psychotherapeutic interventions with adolescents. Being aware of externalizing behavior and increasing the adolescents’ own agency during the assessment could strengthen commitment and result in the adolescent benefiting more from treatment.

## Introduction

In the past three decades there has been an increasing amount of clinical trials yielding a high level of evidence supporting the benefits of psychotherapeutic interventions for a wide range of mental disorders in children and adolescents (La Greca et al., 2009; Weisz et al., 2017). Most evidence-based psychotherapies focus on single conditions, but in clinical practice majority of patients suffer from psychiatric comorbidity (Riosa et al., 2011). Yet evidence of the effectiveness of psychotherapeutic interventions in naturalistic settings or for adolescents with psychiatric comorbidity is still scarce. In a review of current evidence on youth psychotherapy, Weisz et al. (2014) discovered that there were clinically referred patients involved in only 2.1% of the samples in a meta-analysis concerning randomized controlled trials (RCT’s) of child and adolescent psychotherapy. Differences between academic and clinical settings in the nature of therapy, patient characteristics, and administration of research emphasize the need to increase research on effectiveness in clinical service settings in order to increase the generalizability and external validity of the evidence (Weisz et al., 2005; Rich et al., 2014).

Even if strong evidence shows that psychotherapeutic interventions are effective in treating mental disorders in adolescence, no treatment for any disorder is universally effective for all patients, and the understanding of what works for whom and why is far from clear and the evidence on which factors influence successfulness of the treatment is not consistent (La Greca et al., 2009; Nilsen et al., 2012).

Some of these factors are outcome predictors, which are defined as characteristics assessed at baseline which influence the treatment outcome independently of treatment modality and have a major effect but no interaction effect on treatment outcome (Hinshaw, 2007; La Greca et al., 2009). There are several ways to group predictors, such as patient characteristics, family characteristics, clinical characteristics, psychological characteristics, treatment characteristics, or therapist characteristics (Nilsen et al., 2012; de Haan et al., 2013). In many studies the predictors have not been grouped or the groups overlap in different studies. In systematic reviews, the findings concerning youths show mainly no significant associations between demographic or clinical factors with treatment outcome, but there are some indications for baseline symptom severity, comorbidity, intelligence quotient, parents’ mental health, and form of treatment (Hinshaw, 2007; Nilsen et al., 2012). The dose–effect relationship is increasingly being studied with adults, but with adolescents the research is limited, and results are mixed. Target and Fonagy (1994) found length of treatment to predict treatment outcome for youths, but Salzer et al. (1999) and Bachmann et al. (2010) found no general dose–effect relationship based on the number of sessions.

Unfortunately not all adolescents benefit from psychotherapeutic interventions or even give the treatment an opportunity to be effective. Seeking help, admitting having psychological problems, and engaging in psychotherapy may conflict with an adolescent’s age appropriate desire for autonomy, which can be an obstacle for commitment to therapy (Oetzel and Scherer, 2003). However, adolescents are not commonly used as informants in dropout studies, parents or therapists are instead, which highlights the need to focus on adolescents themselves (de Haan et al., 2013).

Among youths receiving special services for mental disorders, the treatment dropout rates are found to be as high as 28–75% (La Greca et al., 2009; Pellerin et al., 2010; de Haan et al., 2013). The majority of studies on dropout for youth psychotherapy are RCT studies, where premature termination rates are lower than in naturalistic studies (de Haan et al., 2013).

Treatment non-completers have been found to differ from completers in a variety of patient, family, sociodemographic, and clinical variables in several studies, but the evidence is mixed. More severe symptoms have been found to predict treatment dropout in some studies (Pellerin et al., 2010), but in other studies antisocial behavior has been found to be the only significant predictor (O’Keeffe et al., 2018). Studies of sociodemographic or patient- and family-related variables have resulted in mixed findings (O’Keeffe et al., 2018). Two treatment-related variables – reduction in alliance and higher level of missed sessions – have shown promising evidence of predicting dropout (O’Keeffe et al., 2018). Even if the findings vary across different study designs and dropout definitions, there are some variables that seem robust predictors of treatment dropout in youths. Among the most important predictors are having more externalizing problems, lower perceived relevance of treatment, and the form of therapy (de Haan et al., 2013).

As engagement to treatment is undoubtedly relevant to be able to benefit from it, it is important to look for reasons why patients might not be satisfied or willing to engage to treatment. Expectations of treatment has been one of the undervalued elements of psychotherapy research especially in adolescents, even if it has been recognized as one of the key elements for change (Greenberg et al., 2006; Wampold, 2015; Weitkamp et al., 2017). One way to define patients’ expectations of their own role in psychotherapy can be examined by looking at the locus of control. It refers to a person’s belief that the consequence (e.g., getting better) depends either on one’s own efforts (internal locus of control) or is controlled by external factors such as chance or powerful others (external locus of control) (Rotter, 1966; Levenson, 1973). A link between high internality and positive outcome has been found, but control expectancy measures in a psychotherapy context are still rare (Delsignore and Schnyder, 2007).

Most of the few studies concerning treatment expectations in adolescents have been retrospective, which may lead to the results being affected by the experiences of treatment (Midgley et al., 2016). Research into adolescents’ expectations of therapy has also been hindered by the lack of measures developed specifically for adolescents (Midgley et al., 2016). Prior to treatment, adolescents seem to expect the therapist to have a strong role in the therapy and to not to have to put in much effort themselves, which may lead to ruptures and premature termination of treatment (Midgley et al., 2016; Weitkamp et al., 2017). Lewis et al. (2009) reported that adolescents who reported high action orientation responded best to treatment regardless of its modality. According to Philips et al. (2007), youths who terminated therapy prematurely were reported to be more distancing (i.e., in denial, avoidance, and neglecting personal responsibility) than approaching (i.e., taking ownership and facing problems), indicating the importance of pretreatment attitudes for therapy commitment similar to the studies on control expectancies with adults.

The aim of this study was to examine outcomes of psychotherapeutic interventions in a 1-year follow-up in a naturalistic setting among adolescent psychiatric outpatients and to explore the predictors of treatment outcome. Based on previous literature patient-related sociodemographic variables (age, gender), clinical variables (comorbidity, type of symptoms, functioning, symptom severity at baseline), treatment-related variables (form of treatment, frequency), and psychological variables (locus of control) were chosen to be tested as possible predictors.

We expected that psychotherapeutic interventions would be effective treatments for mental disorders also with clinically referred adolescents, and to find baseline symptom severity, comorbidity, frequency and form of treatment to be predictors of outcome. In addition, we expected that symptom severity, functioning, externalizing behavior, form of treatment and adolescents’ expectations of their own role in treatment would be related to treatment dropout.

## Materials and Methods

### Design and Procedure

The adolescents were referred to psychotherapeutic interventions conducted by private practitioners from secondary care psychiatric outpatient clinics for adolescents. The study was conducted as part of ordinary follow-up meetings at the outpatient unit remaining responsible from the overall treatment. The participants filled in the self-assessment forms after completing the assessment period for the therapy and again after 3, 6, and 12 months of treatment. The study design, procedure and preliminary results of the effectiveness for the first 3-month treatment period have been published in more detail in Gergov et al. (2015).

This study was accepted by the Ethics Committee of the Helsinki and Uusimaa Hospital District (276/13/03/03/2011), granted by the pertinent institutional authorities of the hospital (704/13/2011), and conducted at the Division of Adolescent Psychiatry in the Department of Psychiatry in Helsinki University Hospital in Finland. All participants and their legal guardians provided their written informed consent to participate after receiving verbal and written information about the study. Refusal did not affect the treatment the adolescents received, and the adolescent participating had the option to intercept the treatment at any point.

### Participants

The participants were 13- to 15-year-old adolescents (mean = 14.22, *SD* = 0.73; 65.5% girls). Altogether, 61 (70.7% of approached patients) adolescents referred to psychotherapeutic interventions between 1st of February 2012 to 31st of January 2014 agreed to participate in the study, with 59 of them starting the intervention and 58 filling in the questionnaires prior to treatment. Sociodemographic variables were reported and psychiatric diagnoses using the ICD-10 classification (World Health Organization (WHO), 1992) were assessed by psychiatrists responsible for the patients’ care. Major diagnostic groups were F40-49: Neurotic, stress-related and somatoform disorders (43.1%), F30-39: Mood disorders (27.6%), and F90-98: Behavioral and emotional disorders (20.7%). There were no exclusion criteria for the study. The sample did not significantly differ in background variables from the average adolescent patient population receiving publicly funded psychotherapeutic interventions in the Helsinki University Hospital (Gergov et al., 2015). The sample characteristics are presented in Table 1.

**TABLE 1:** Demographics and sample characteristics of the participants (*n* = 58).

Variables	Form of the intervention		Frequency of the intervention		Total (*n* = 58)
		
	Psychotherapy (*n* = 37)	Art/occupational therapy (*n* = 21)	Once a week or more seldom (*n* = 29)	Twice a week (*n* = 29)	
**Sociodemographic variables**					
Age, mean (*SD*)	14.22 (0.75)	14.24 (0.70)	14.24 (0.74)	14.21 (0.73)	14.22 (0.73)
Gender: female	25 (67.6)	12 (31.6)	19 (65.5)	19 (65.5)	38 (65.5)
Living with biological parents	29 (78.4)	18 (85.7)	22 (75.9)	25 (86.2)	47 (81.0)
**Clinical variables**					
Previous mental health contact	18 (48.6)	12 (57.1)	15 (51.7)	15 (51.7)	30 (51.7)
Length of psychiatric treatment before the index intervention, months, mean (*SD*)	7.86 (5.44)	12.05 (5.83)	10.59 (5.81)	8.17 (5.83)	9.38 (5.89)
Psychotropic medication	23 (62.2)	15 (71.4)	19 (65.5)	19 (65.5)	38 (65.5)
Psychiatric comorbidity	16 (43.2)	12 (57.1)	15 (51.7)	13 (44.8)	28 (48.3)
Type of symptoms (externalizing)	7 (18.9)	8 (38.1)	11 (37.9)	4 (13.8)	15 (25.9)
Diagnostic groups according to the principal diagnoses (ICD-10)					
*F30-39 Mood disorders*	10 (27.0)	6 (28.6)	6 (20.7)	10 (34.5)	16 (27.6)
*F40-49 Neurotic, stress-related and somatoform disorders*	17 (45.9)	8 (38.1)	13 (44.8)	12 (41.4)	25 (43.1)
*F50-59 Behavioral syndromes associated with physiological disturbances and physical factors*	2 (5.4)	0 (0.0)	2 (6.9)	0 (0.0)	2 (3.4)
*F80-89 Disorders of psychological development*	2 (5.4)	1 (4.8)	1 (3.4)	2 (6.9)	3 (5.2)
*F90-98 Behavioral and emotional disorders*	6 (16.2)	6 (28.6)	7 (24.1)	5 (17.2)	12 (20.7)
Symptom severity and level of functioning at baseline					
*C-GAS, mean (*SD*)*	53.83 (8.05)	53.95 (7.53)	52.48 (9.67)	55.07 (5.64)	53.87 (7.80)
*BDI total score, mean (*SD*)*	14.43 (12.86)	14.14 (15.15)	11.86 (13.21)	16.79 (13.76)	14.33 (13.60)
*CORE-OM total score, mean (*SD*)*	1.33 (0.77)	1.24 (0.82)	1.10 (0.79)	1.49 (0.75)	1.30 (0.79)
*CORE-OM well-being, mean (*SD*)*	1.69 (0.98)	1.56 (1.04)	1.43 (1.04)	1.86 (0.92)	1.64 (0.99)
*CORE-OM problems/symptoms, mean (*SD*)*	1.54 (0.97)	1.26 (1.07)	1.17 (0.99)	1.71 (0.96)	1.44 (1.01)
*CORE-OM life functioning, mean (*SD*)*	1.41 (0.76)	1.47 (0.77)	1.30 (0.80)	1.57 (0.70)	1.43 (0.76)
*CORE-OM risk/harm, mean (*SD*)*	0.49 (0.68)	0.46 (0.69)	0.36 (0.62)	0.61 (0.72)	0.48 (0.68)
*SDQ total score, mean (*SD*)*	14.00 (5.32)	13.67 (6.55)	13.38 (6.01)	14.38 (5.52)	13.88 (5.74)
*SDQ emotional symptoms, mean (*SD*)*	5.11 (2.74)	4.43 (2.96)	4.17 (2.90)	5.55 (2.59)	4.86 (2.81)
*SDQ conduct problems, mean (*SD*)*	2.03 (1.62)	2.38 (1.83)	2.24 (1.62)	2.07 (1.79)	2.16 (1.69)
*SDQ hyperactivity, mean (*SD*)*	3.95 (1.97)	4.48 (2.62)	4.66 (2.35)	3.62 (1.99)	4.14 (2.22)
*SDQ peer problems, mean (*SD*)*	3.19 (2.04)	2.62 (1.80)	2.66 (1.95)	3.31 (1.95)	2.98 (1.96)
*SDQ prosocial behavior, mean (*SD*)*	7.92 (2.48)	7.52 (1.66)	7.97 (2.56)	7.59 (1.82)	7.76 (2.21)
**Treatment-related variables**					
Form of treatment (psychotherapy)			15 (51.7)	22 (75.9)	37 (63.8)
Frequency (twice a week)	15 (40.4)	7 (33.3)			29 (50.0)
Parental guidance involved in treatment	34 (91.9)	19 (90.5)	25 (86.2)	28 (96.6)	53 (91.4)
**Psychological variables**					
External locus of control	8 (22.2)	4 (20.0)	7 (25.0)	5 (17.9)	12 (21.4)

*Unless otherwise indicated, data are expressed as number (percentage).*

*C-GAS, Children’s Global Assessment Scale; BDI, Beck Depression Inventory; CORE-OM, Clinical Outcomes in Routine Evaluation – Outcome Measure; SDQ: Strengths and Difficulties Questionnaire.*

### Treatment

As it is a naturalistic sample, the 47 therapists participating in the study represented several different psychotherapeutic approaches. In Finland the training for different types of treatment modalities is regulated, and all psychotherapists and occupational therapists, as well as most art therapists, are legalized health care professionals that have been accepted as private health care practitioners by national authorities. All therapists participating in the study were trained and certified for the form of therapy they provided. No standard treatment protocol was demanded. The interventions included psychotherapies (*n* = 37, 63.8%) including psychodynamic (*n* = 22), cognitive (*n* = 5), crisis- and trauma-focused (*n* = 3), and family therapy (*n* = 7); and art and occupational therapies (*n* = 21, 36.2%) including music (*n* = 10), art (*n* = 5), occupational (*n* = 4), and riding therapy (*n* = 2). One therapist treated four patients, one therapist had three patients, seven treated two, and the remaining 38 therapists treated one patient each. Based on intra class correlation coefficient, therapist level didn’t significantly explain variation in any treatment outcome (ICC: 0.00 – 0.06). Altogether, 81.0% of the participants received individual therapy, 12.1% family therapy, and 6.9% group therapy. Half of the patients were pre-assigned to receive treatment twice a week, and half to receive treatment once a week or more seldom according to the recommendation of the psychiatrist responsible for the patients care and assigning him/her to the target treatment.

### Measures

#### Outcome Measures

##### Beck Depression Inventory (BDI-21)

Participants completed the Beck Depression Inventory, BDI-21 (Beck et al., 1961), self-report to assess depressive symptoms. The BDI-21 has been widely used in treatment outcome studies in adolescent populations, and it has shown good psychometric properties in multiple studies (Ambrosini et al., 1991). In this study, the internal consistency of the questionnaire also proved to be good (Cronbach’s alpha, α, 0.95).

##### Clinical Outcomes in Routine Evaluation – Outcome Measure (CORE-OM)

Clinical Outcomes in Routine Evaluation – Outcome Measure (Evans et al., 2000) is a pan-theoretical self-report questionnaire measuring psychological distress. Each of the 34 statements is evaluated using a 5-point Likert scale using scores from 0 to 4, so the total score can range from 0 to 136. The CORE-OM comprises four scales: subjective well-being (four items), problems/symptoms (12 items), life functioning (12 items), and risk/harm (six items). The score for each scale is the mean total score of the items. The CORE–OM has shown to be a reliable and valid instrument with good sensitivity to change (Evans et al., 2002). The internal consistency of the questionnaire in this study had a α of 0.96.

#### Other Measures

##### Strengths and Difficulties Questionnaire (SDQ)

The SDQ is a 25-item self-assessment measure of psychosocial symptoms in children and adolescents (Goodman, 1997). Along the total score, an internalizing scale including emotional symptoms and peer problems, and an externalizing scale including conduct problems and hyperactivity can be formed. The SDQ has been widely used among adolescents, and its reliability and validity have been demonstrated to be good (Goodman, 2001; Muris et al., 2003). In this study, the SDQ was used as self-report at baseline to identify the type of symptoms (externalizing/internalizing), and the internal consistency (α) of the questionnaire was 0.78.

##### Questionnaire on Control Expectancies in Psychotherapy (Fragebogen zu therapiebezogenen Kontrollerwartungen, TBK)

The TBK assesses patients’ control expectancies related to the psychotherapy process (Delsignore et al., 2006). The TBK includes 18 items forming the dimensions of internal and external control, and has shown good construct and concurrent validity upon development (Delsignore and Schnyder, 2007). The latter includes items related to therapist control and chance. To our knowledge, the questionnaire has so far been used only with adults, also including the Finnish translation (Pihlaja, 2013). In this study, the TBK was used to identify the patients’ locus of control at baseline. The internal consistency (α) for the dimension of the internal locus of control was 0.61, and for the external locus of control α was 0.74.

### Data Analyses

Statistical analyses were carried out using SPSS version 25. The internal consistency of the measures was tested for the whole sample using α. A α-score over 0.60 was considered acceptable (Taber, 2018).

The significance of change in the symptom measures between the baseline and three time points (3, 6, 12 months) was assessed by a linear mixed model. The comparison between the different subgroups of form and frequency of therapy and patients’ own role expectations (locus of control) was also conducted with a linear mixed model. There was no statistically significant relationship between the form of treatment and the frequency of sessions (*p* = 0.06), so they could be examined separately in the analyses.

The difference in change in the symptom measures at different time points was compared between the adolescents who dropped out in the first 12 months and the adolescents who continued the therapy as planned by linear mixed model. Error covariance was set to unstructured in all analyses conducted with the linear mixed models. The analysis for predicting the outcome and therapy dropout in the first 12 months was conducted by separate logistic regression analysis. Considering the dropouts, statistical significance was determined based on 5,000 bootstrapped bias-corrected resamples. The differences between the subgroups of predictors at baseline were examined using an independent samples *t*-test. The possible effects of age, gender, and psychotropic medication was controlled in all analyses. The adolescents who declined to continue their participation in the study in the first 12 months (*n* = 3) were not included in the analysis of predicting the treatment outcome or therapy dropouts.

The level of significance was defined as *p* < 0.05. Effect sizes are reported by using marginal *R*^2^ for all fixed effects (Nakagawa and Schielzeth, 2013; Johnson, 2014; Nakagawa et al., 2017). Effect size estimation was carried out using the MuMIn package (Barton, 2019) with R software version 3.5.1 (R Core Team, 2020). The magnitude of *R*^2^ was interpreted as a “small,” “medium,” and “large” effect with cutoff points of 0.02, 0.13, and 0.26, respectively (Cohen, 1988). Odds Ratios were transformed to *R*^2^ according to Lenhard and Lenhard (2016).

Power calculations for linear mixed models were done by simulation (500 simulation per analysis), using simr-package (Green and MacLeod, 2016) in R-software version 4.0.3 (R Core Team, 2020). We concluded that using the available sample size of 58 we could only detect large effect sized as statistically significant with 80% power. Also observed power was calculated as a benchmark for future research and as expected ranged from 49–74% for statistically significant results and from 0–52% for insignificant results. Similarly when evaluating treatment dropout using logistic regression analysis with the sample size of 58 medium effect sizes (Odds ratios over 3.5) could be detected as statistically significant based on *a priori* power analysis with G^∗^Power version 3.1.9.2 software (Faul et al., 2007). Observed power was also calculated as a benchmark for future research and as expected ranged from 96 to 97% for statistically significant results and from 5 to 76% for insignificant.

## Results

### Effectiveness on Symptom and Psychological Distress Reduction

Symptoms reduced and psychological distress decreased over the course of therapy and follow-up as indicated by BDI-21 [*F*(3,49) = 4.17, *p* = 0.01, full model *R*^2^ = 0.19], CORE-OM total score [*F*(3,47) = 4.21, *p* = 0.01, full model *R*^2^ = 0.15], CORE-OM well-being [*F*(3,47) = 5.86, *p* < 0.01, full model *R*^2^ = 0.21], CORE-OM problems/symptoms [*F*(3,47) = 3.28, *p* = 0.03, full model *R*^2^ = 0.13], and CORE-OM life functioning [*F*(3,47) = 3.68, *p* = 0.02, full model *R*^2^ = 0.12]. The reduction was more significant in the first 6 months than after that. Changes between different time points in all outcome measures were analyzed and are presented in Table 2. After excluding the treatment dropouts from the analyses, the significance of the effect of time on treatment outcomes weakened, but the interpretation of the results did not change.

**TABLE 2:** Change between all time points in the outcome measures (*n* = 58).

Time points compared		BDI-21 total score			CORE-OM total score			CORE-OM well- being			CORE-OM problems/symptoms			CORE-OM life functioning			CORE-OM risk/harm		
							
		Mean Diff.	*SE*	*p*	Mean Diff.	*SE*	*p*	Mean Diff.	*SE*	*p*	Mean Diff.	*SE*	*p*	Mean Diff.	*SE*	*p*	Mean Diff.	*SE*	*p*
Baseline	3 months	2.41	1.02	0.021*	0.14	0.08	0.086	0.33	0.10	0.002*	0.07	0.10	0.510	0.13	0.09	0.135	0.13	0.07	0.077
	6 months	4.12	1.20	0.001*	0.32	0.09	0.001*	0.54	0.13	< 0.001*	0.32	0.11	0.005*	0.33	0.10	0.002*	0.16	0.08	0.059
	12 months	1.83	1.63	0.267	0.20	0.11	0.076	0.33	0.14	0.022*	0.13	0.13	0.337	0.27	0.12	0.029*	0.08	0.10	0.412
3 months	6 months	1.71	1.07	0.117	0.19	0.08	0.018*	0.21	0.11	0.070	0.25	0.10	0.015*	0.20	0.09	0.030*	0.03	0.05	0.530
	12 months	–0.58	1.49	0.699	0.07	0.10	0.521	0.00	0.14	0.985	0.06	0.12	0.595	0.14	0.10	0.173	–0.04	0.10	0.684
6 months	12 months	–2.29	1.56	0.149	–0.12	0.11	0.254	–0.21	0.13	0.133	–0.19	0.13	0.151	–0.06	0.11	0.598	–0.07	0.08	0.401

Increase in mean difference refers to symptom reduction.

*Significant at *p* < 0.05 level.

*BDI, Beck Depression Inventory; CORE-OM, Clinical Outcomes in Routine Evaluation – Outcome Measure.*

### Predictors of Treatment Outcome

The sociodemographic and clinical variables or the locus of control did not predict the outcome on any of the symptom or psychological distress scales (*p*-values > 0.05). Different forms of therapy (psychotherapy vs. art and occupational therapies) did not differ significantly from each other in any of the outcome measures (*p*-values > 0.05) when looking at the change between baseline and different time points. Frequency of treatment sessions (twice a week vs. once a week or more seldom) was related to treatment outcome on most of the measured scales: Frequency of sessions moderated the change in BDI-21 (*p* = 0.04, *R*^2^ = 0.20), CORE-OM total score (*p* = 0.02, *R*^2^ = 0.17), CORE-OM well-being (*p* = 0.05, *R*^2^ = 0.23), CORE-OM problems/symptoms (*p* = 0.01, *R*^2^ = 0.15), and CORE-OM life functioning (*p* = 0.04, *R*^2^ = 0.14) such that there was significantly more change and the change happened earlier when therapy was more frequent. Results of interaction effects between time and predictor variables for all outcome measures are presented in Table 3.

**TABLE 3:** Predictors of treatment outcome based on interactions between time and the outcome measures.

Predictor	BDI total score						CORE-OM total score						CORE-OM well-being						CORE-OM problems/symptoms						CORE-OM life functioning						CORE-OM risk/harm					
						
	*df1*	*df2*	*F*	*p*	*R*^2^	*Obs. pw*	*df1*	*df2*	*F*	*p*	*R*^2^	*Obs. pw*	*df1*	*df2*	*F*	*p*	*R*^2^	*Obs. pw*	*df1*	*df2*	*F*	*p*	*R*^2^	*Obs. pw*	*df1*	*df2*	*F*	*p*	*R*^2^	*Obs. pw*	*df1*	*df2*	*F*	*p*	*R*^2^	*Obs. pw*
**Sociodemographic variables**																																				
Age	3	48.19	0.13	0.944	0.19	0.08	3	45.66	0.22	0.884	0.15	0.10	3	45.75	1.01	0.395	0.22	0.21	3	45.66	0.02	0.995	0.13	0.02	3	47.40	1.42	0.250	0.13	0.36	3	49.42	0.91	0.442	0.09	0.35
Gender	3	48.29	1.04	0.383	0.20	0.00	3	45.75	1.17	0.331	0.16	0.00	3	45.57	1.21	0.318	0.21	0.00	3	46.02	1.15	0.340	0.14	0.00	3	47.23	1.37	0.264	0.14	0.00	3	49.47	1.64	0.191	0.10	0.00
**Clinical variables**																																				
Psychotropic medication	3	48.89	1.61	0.199	0.20	0.52	3	46.71	1.71	0.179	0.15	0.53	3	46.68	0.59	0.623	0.21	0.22	3	46.87	1.48	0.231	0.13	0.45	3	47.92	1.38	0.262	0.12	0.46	3	50.19	1.23	0.307	0.09	0.31
Comorbidity	3	48.00	1.38	0.259	0.20	0.00	3	45.89	0.65	0.588	0.15	0.00	3	45.47	0.91	0.443	0.22	0.00	3	46.06	0.87	0.462	0.13	0.00	3	47.30	0.49	0.691	0.13	0.00	3	48.65	0.26	0.856	0.11	0.00
Type of symptoms (externalizing)	3	48.85	0.18	0.907	0.19	0.00	3	46.31	0.03	0.993	0.16	0.00	3	45.93	0.09	0.965	0.22	0.00	3	46.47	0.29	0.836	0.14	0.00	3	47.95	0.41	0.747	0.13	0.00	3	50.07	0.14	0.933	0.08	0.00
C-GAS baseline	3	45.08	0.69	0.560	0.19	0.00	3	44.06	1.24	0.306	0.16	0.00	3	44.07	1.28	0.293	0.23	0.00	3	43.78	0.73	0.541	0.15	0.00	3	44.39	3.21	0.032	0.14	0.00	3	44.61	0.33	0.800	0.10	0.00
BDI total score baseline	2	45.03	0.79	0.459	0.71	0.00	2	43.67	0.23	0.795	0.52	0.00	2	44.49	0.54	0.589	0.46	0.00	2	43.55	0.15	0.860	0.47	0.00	2	44.24	0.14	0.867	0.45	0.00	2	44.20	1.49	0.237	0.40	0.00
CORE-OM total score baseline	2	45.07	0.78	0.463	0.62	0.00	2	43.75	0.24	0.786	0.58	0.00	2	44.80	0.75	0.479	0.52	0.00	2	43.71	0.16	0.855	0.55	0.00	2	44.04	0.06	0.941	0.52	0.00	2	43.82	1.52	0.230	0.39	0.00
CORE-OM well-being baseline	2	44.52	0.59	0.558	0.55	0.00	2	43.14	0.39	0.683	0.51	0.00	2	44.25	0.92	0.407	0.55	0.00	2	43.44	0.52	0.597	0.44	0.00	2	43.12	0.27	0.768	0.45	0.00	2	43.24	1.01	0.373	0.33	0.00
CORE-OM problems/symptoms baseline	2	45.14	0.77	0.468	0.56	0.00	2	43.97	0.18	0.834	0.58	0.00	2	44.80	0.66	0.521	0.49	0.00	2	43.76	0.08	0.922	0.61	0.00	2	44.47	0.12	0.884	0.46	0.00	2	43.62	1.33	0.274	0.34	0.00
CORE-OM life functioning baseline	2	44.94	1.15	0.326	0.48	0.00	2	43.70	0.50	0.610	0.45	0.00	2	44.49	1.05	0.358	0.42	0.00	2	43.83	0.21	0.815	0.36	0.00	2	44.22	0.38	0.685	0.49	0.00	2	43.94	1.11	0.338	0.26	0.00
CORE-OM risk/harm baseline	2	45.88	0.45	0.639	0.59	0.00	2	44.10	0.87	0.427	0.45	0.00	2	44.58	0.43	0.656	0.41	0.00	2	44.08	0.70	0.500	0.39	0.00	2	44.43	1.52	0.230	0.36	0.00	2	44.06	1.63	0.207	0.57	0.00
**Treatment-related variables**																																				
Form of treatment	3	48.58	0.14	0.935	0.19	0.04	3	45.99	0.17	0.917	0.16	0.05	3	45.69	0.42	0.741	0.21	0.12	3	46.14	0.53	0.661	0.14	0.12	3	47.32	0.45	0.721	0.13	0.15	3	48.99	0.30	0.826	0.09	0.10
Frequency of treatment	3	48.70	3.07	0.036*	0.20	0.58	3	46.37	3.57	0.021*	0.17	0.74	3	45.41	2.74	0.054*	0.23	0.69	3	47.21	4.13	0.011*	0.15	0.78	3	47.97	3.10	0.035*	0.14	0.49	3	48.72	0.67	0.577	0.09	0.24
**Psychological variables**																																				
Locus of control	3	46.69	0.28	0.837	0.18	0.00	3	43.75	0.98	0.410	0.15	0.00	3	43.92	1.08	0.366	0.21	0.00	3	43.91	0.50	0.683	0.14	0.00	3	45.45	1.64	0.194	0.12	0.00	3	48.72	0.16	0.921	0.09	0.00

*Significant at *p* < 0.05 level.

*C-GAS, Children’s Global Assessment Scale; BDI, Beck Depression Inventory; CORE-OM, Clinical Outcomes in Routine Evaluation – Outcome Measure; Obs.pw, Observed power.*

### Predictors of Treatment Dropout

There were 10 treatment dropouts (17.2%), none occurring in the first 3 months of treatment. In the first 3 months symptoms decreased significantly more among adolescents who dropped out from treatment between three and 12 months than among those who didn’t drop out in the CORE-OM total score [*t*(53) = 2.21, *p* = 0.03], CORE-OM well-being [*t*(53) = 2.56, *p* = 0.01], and CORE-OM life functioning [*t*(53) = 2.44, *p* = 0.02].

Adolescents with higher levels of externalizing problems at baseline were at higher risk of dropping out (*p* = 0.04, OR = 4.00, *R*^2^ = 0.13). The result remained when all other symptom measure subscales were controlled for. Patients’ own role expectations of responsibility for change in treatment significantly predicted dropout (*p* = 0.04, OR = 4.23, *R*^2^ = 0.14) so that adolescents who rated the locus of control external more likely dropped out than adolescents with a higher internal locus of control. None of the other variables defined as possible predictors reached statistical significance on predicting treatment dropout. The effects of all predictors of dropout based on separate logistic regression analysis are shown in Table 4.

**TABLE 4:** Predictors of treatment dropout (*n* = 10) based on separate logistic regression analysis.

Predictor	*p*	*OR*	*95% CI for OR*	*R*^2^	Obs.pw
**Sociodemographic variables**					
Age	0.664	1.27	0.41–6.10	<0.01	0.11
Gender (female)	0.321	0.50	0.00–2.28E + 08	0.04	0.49
**Clinical variables**					
Psychotropic medication (yes)	0.908	1.05	0.09–7.48E + 08	<0.01	0.05
Comorbidity (yes)	0.838	1.14	0.36–3.75	<0.01	0.06
Type of symptoms (externalizing)	0.038*	4.00	1.13–16.01	0.13	0.96
C–GAS baseline	0.165	1.05	0.98–1.21	<0.01	0.05
BDI total score baseline	0.456	1.02	0.96–1.09	<0.01	0.05
CORE–OM total score baseline	0.293	1.52	0.59–4.74	0.01	0.23
CORE–OM well–being baseline	0.219	1.46	0.75–3.31	0.01	0.19
CORE–OM problems/symptoms baseline	0.665	1.15	0.60–2.33	<0.01	0.07
CORE–OM life functioning baseline	0.139	1.81	0.73–5.97	0.03	0.39
CORE–OM risk/harm baseline	0.568	1.29	0.40–4.07	<0.01	0.12
**Treatment–related variables**					
Form of treatment (psychotherapy)	0.067	0.30	0.09–0.81	0.10	0.76
Frequency of treatment (twice a week)	0.610	1.43	0.32–8.72	<0.01	0.18
**Psychological variables**					
External locus of control	0.039*	4.23	0.66–27.33	0.14	0.97

*Significant at *p* < 0.05 level.

*C-GAS, Children’s Global Assessment Scale; BDI, Beck Depression Inventory; CORE-OM, Clinical Outcomes in Routine Evaluation – Outcome Measure; Obs.pw, Observed power.*

## Discussion

The first aim of this study was to examine the effectiveness of psychotherapeutic interventions for adolescents in a naturalistic setting to increase the generalizability of the evidence for youth psychotherapy. The results support the scarce evidence that psychotherapeutic interventions are effective also with clinically referred adolescents. The effect sizes were on a medium level on both outcome measures and in most of the subscales. Symptoms and psychological distress reduced more in the first 6 months of treatment and remained quite stable during the longer treatment period, which is also in line with previous studies (Bachmann et al., 2010). This might also imply that adolescents improve faster and require less therapy to reach significant change than adults, as Asay et al. (2002) have concluded. A further study comparing different age groups would be needed for stronger conclusions. As Kazdin (1996) emphasizes, there can be different goals and possible benefits of treatment, and changes occur over the course of treatment in phases. Some of the goals might be gained earlier (e.g., subjective well-being or symptom reduction) than others (e.g., changes in life functioning or more enduring characteristics).

Our second aim was to study whether sociodemographic, clinical or treatment-related variables and patients’ role expectations about therapy predict the outcome. Previous studies of child and adolescent psychotherapy mostly do not support the relevance of demographic or clinical factors for predicting treatment outcome, despite some indications for baseline symptom severity or comorbidity being possible predictors (Hinshaw, 2007; Nilsen et al., 2012). Our findings also support the view that sociodemographic or clinical variables are not very strong predictors of outcome.

For treatment-related variables, the form of treatment was not a significant predictor of any of the measured outcome variables. Art and occupational therapies were found to be as effective as psychotherapies, which may indicate the importance of common factors also for adolescents (Karver et al., 2006; Miller et al., 2008; Wissow et al., 2008; Kelley et al., 2010; Weisz et al., 2017). Frequency of therapy sessions was the most important predictor of treatment outcome. Patients receiving therapy twice a week had better outcomes than those receiving treatment once a week or more seldom on most of the outcome measure scales. The effect sizes of 0.20 or above in depressive symptoms and well-being are actually quite high for real-world data since it means that more than 20% of the variation in the outcome was explained by the predictor (Cohen, 1988). The finding is in line with previous findings (Angold et al., 2000) reporting that the number of treatment sessions is related to symptom reduction. The finding supports the need for more intensive treatment which might also reduce the length of treatment needed.

The adolescents’ own role expectations did not predict treatment outcome significantly, which was a bit surprising considering the previous evidence of the significance of the effect of patients’ own expectations on the outcome (Lewis et al., 2009). It is possible, that over the course of the treatment, adolescents’ role expectations change, and they accept more active role. On the other hand, since adolescents tend to expect the therapist to have a strong role in therapy (Weitkamp et al., 2017), the therapists might be more actively taking the lead of the process than with adults, which may result that the effect of patients own expectations is less significant predictor of outcome with adolescents. Also therapeutic alliance could be an important mediator explaining the relation between patients’ own expectations and treatment outcome, so this relation would be an important question for further research.

Finally, we focused on risk factors for treatment dropout in adolescents. The exploratory approach in this naturalistic study sets a benchmark for further clinical trials on treatment dropout for adolescents, but the results must be considered as referential since the statistical power was low due to small sample size. Looking at the clinical predictors, if the adolescent had mainly externalizing symptoms, he/she was more likely to drop out. This is in line with previous findings (Kazdin, 1996; Pellerin et al., 2010; de Haan et al., 2013; O’Keeffe et al., 2018) pointing out that externalizing problems and disruptive or antisocial behavior are among the strongest predictors of treatment dropout in adolescents. In these cases, the therapist should be cautious about the higher risk of dropout and focus more carefully on keeping the adolescent in treatment. As in most studies concerning treatment dropout in adolescents (de Haan et al., 2013), no other clinical or treatment-related variables were found to significantly predict dropping out.

Adolescents reporting higher level of external locus of control had a significantly higher risk of dropout than adolescents who expected their own role to be more active. This result supports the evidence from Weitkamp et al. (2017) stating that paying attention to adolescents’ role expectations and supporting them toward taking more responsibility for change could prevent later treatment dropout. The assessment and research on adolescents’ expectations of their own role in obtaining change in psychotherapeutic interventions should focus on the time before the treatment starts in order to be able to use the information in the clinical context and prevent adolescents at higher risk from dropping out.

Since treatment dropout rates for adolescents are usually found to be relatively high, therapy effects should be gained early to make sure that most of the adolescents stay in treatment long enough to benefit from it. In our study, there were no treatment dropouts in the first 3 months, which is quite uncommon especially in naturalistic settings. This might suggest that the participants were well prepared for psychotherapeutic treatment, as they all had previously received treatment in adolescent psychiatric outpatient care.

As Kazdin (1996) and de Haan et al. (2013) have stated, some patients can be considered successful terminators even if they terminate the treatment earlier than planned, because sufficient improvement in their mental health was achieved in a shorter duration than expected. This seems to be the case also in our study. Concerning the possible interpretation that adolescents improve faster and need less therapy to reach significant change in symptom reduction than adults, it is important to assess the goals of treatment individually before making a referral to psychotherapy. For other types of desired outcomes than symptom reduction, longer treatment might be needed.

### Strengths and Limitations

The strength of this study is its naturalistic setting, which allows the results to be generalized to clinical practice. Another strength of this study was that the treatments were independent as most of the therapists treated only one of the patients. To evaluate possible therapist effects a larger sample would be needed. Unfortunately the naturalistic setting of the study resulted in a relatively small sample size, which limited statistical power in the analyses and increased the risk that some of the results might be caused by type 1 error. Also, some of the non-significant results could be due to lack of statistical power, meaning that some of the possible predictors tested could be important even if they did not reach statistical significance in this study. The heterogeneity of the sample might cause more variance in measured variables.

All diagnoses were not based on structured clinical interviews, such as K-SADS-PL, but instead to psychiatrists’ evaluation based on clinical interviews of adolescents and their legal guardians, and self-report questionnaires. Since it was a transdiagnostic study using diagnosis only as a descriptive baseline characteristic, the assessment for diagnosis was considered to be satisfactory.

The BDI-21 and SDQ have been widely used among adolescents and have demonstrated good psychometric properties in this age group. The CORE-OM and TBK have been developed for adults, and as yet there are no appropriate studies concerning their psychometric properties in youth populations available. In this study the internal consistency (α) for the CORE-OM was good, and for the dimensions in TBK acceptable, but not very high. Further research on the psychometric properties and suitability of the measures for adolescents is needed. A youth version of the CORE-OM has also been published (YP-CORE; Twigg et al., 2009), but the Finnish version (Gergov et al., 2017) was not available at the beginning of this study.

The number of dropouts in this study was lower than in most studies concerning psychotherapeutic interventions for adolescents, so the results presented on treatment dropout should be considered as preliminary, setting a benchmark for further research with larger samples. In this study, we could not examine the reasons why adolescents dropped out. It might be that they dropped out partly because they were not satisfied to the treatment, but perhaps also because they had gained a sufficient reduction in symptoms, as the adolescents who dropped out benefited more from the interventions in the first 3 months in terms of symptom reduction compared with the adolescents who stayed in treatment for the full 12-month period. It is also good to recognize that no single factor may be necessary or sufficient, and an adolescent is most likely to drop out from treatment when multiple risk factors are present (Kazdin, 1996). A further limitation of the study is that we could not study the possible link between engaging to the treatment in terms of the number or percentage of the sessions the adolescents attended and the outcome or dropout from the treatment.

## Conclusion

The results from this study strengthen the evidence of the effectiveness of psychotherapeutic interventions in adolescents in naturalistic settings. In terms of symptom reduction and functioning, the interventions seem to be most effective in the first 6 months, and the results remain quite stable during a longer treatment period. The frequency of treatment sessions was the strongest predictor of good outcome. These results indicate that before referring an adolescent for psychotherapeutic treatment it is important to carefully assess what the main goals for treatment are and base the treatment length recommendation on the goals defined with the patient. It is important to keep in mind that adolescents might need less treatment to gain significant changes than adults and that adolescents also tend to drop out from treatment quite often. Based on our results, it seems that offering more intensive treatment for a shorter period might be the most efficient way to reduce symptoms and increase functioning, but further research is needed to strengthen this conclusion and to study the indications for other types of outcomes and goals of treatment.

As having more externalizing problems seem to drop out more commonly, it is important that therapists are aware of whether this type of clinical risk factors are present, so that they could put more effort into motivating the adolescent and keeping him/her in treatment. Part of the assessment before the therapeutic intervention should also be evaluating the adolescents’ own role expectancies in the treatment process. Increasing the adolescents’ agency in the expected change in treatment already during the assessment period could strengthen the adolescents’ commitment to treatment and increase the likelihood of them benefiting more from it. The treatment plan should also be re-evaluated often to keep the patients committed and to avoid unfair designation of premature termination in case improvement is faster than expected.

## Data Availability Statement

The datasets presented in this article are not readily available because the approval from the Ethics Committee of the Helsinki and Uusimaa Hospital District and the institutional authors of the hospital allow the data to be used only in the study in question, and the participants with their legal guardians have given their permission for this purpose, so the data cannot be shared with third parties. Requests to access the datasets should be directed to VG, vera.gergov@helsinki.fi.

## Ethics Statement

The study was approved by the Ethics Committee of the Helsinki and Uusimaa Hospital District. Written informed consent to participate in this study was provided by the participants and also the participants’ legal guardian.

## Author Contributions

VG has started the research and formulated the design of the study. She has been responsible for gathering and analyzing the data and drafting the manuscript. JLa, MM, and NL have supervised VG, and revised the manuscript several times giving their expertise also by modifying the drafts. MM and NL have also contributed to the study design. JLi has had a significant role on the data analysis and interpreting the results and has been involved especially on writing the methods and results. All the authors have given their approval to publish the study.

## Conflict of Interest

The authors declare that the research was conducted in the absence of any commercial or financial relationships that could be construed as a potential conflict of interest.
